# A cost analysis of reductions in work productivity for MG patients and their caregivers by symptom severity

**DOI:** 10.3389/fpubh.2025.1538789

**Published:** 2025-04-25

**Authors:** Sarah Dewilde, Cynthia Z. Qi, Femke De Ruyck, Sandra Paci, Lucas Van de Veire, Alison Griffiths, Gil I. Wolfe, Renato Mantegazza, Glenn Phillips

**Affiliations:** ^1^Services in Health Economics, Brussels, Belgium; ^2^argenx, Boston, MA, United States; ^3^argenx, Ghent, Belgium; ^4^Department of Neurology, Jacobs School of Medicine and Biomedical Sciences, University at Buffalo/SUNY, Buffalo, NY, United States; ^5^Emeritus, Fondazione IRCCS Istituto Neurologico Carlo Besta, Milan, Italy

**Keywords:** ADAPT, caregivers, costs, efgartigimod, GMG, generalized myasthenia gravis, MyRealWorld-MG, productivity losses

## Abstract

**Introduction:**

Myasthenia Gravis (MG) is a debilitating autoimmune disorder associated with fatigue and weakness in the ocular, respiratory, bulbar and limb muscles. This study evaluates productivity losses for MG patients and their caregivers by MG symptom severity.

**Methods:**

In the multinational MyRealWorld-MG study, 1,049 MG patients and caregivers reported on work productivity (sick leave, reduced working hours, early retirement). Productivity losses were calculated using the average wage per hour. A UK perspective was adopted for the whole sample, and country-specific analyses were conducted for Italy, Spain and the US. The MG-Activities of Daily Living (MG-ADL) score was used to estimate the association between symptom severity and productivity losses, with patients categorized as having mild (0–4), moderate (5-9), or severe (> = 10) symptoms.

**Results:**

In the MyRealWorld-MG study, 36.5% of MG patients reported taking sick leave within the last month and 11.4% reported stopping work (or retiring early) due to MG. Furthermore, 36.0% required caregiver support with 14.6% of caregivers reducing working hours and 13.4% stopping work. Mean productivity losses were £16,630/year for patients and caregivers combined, largely attributable to patient productivity losses (£13,891). Patients with severe MG incurred 3.8 times more productivity losses compared to patients with mild disease. Productivity loss estimates varied between Italy, Spain and the US.

**Conclusion:**

The impact of MG on patients’ and caregivers’ work productivity leads many of them to reduce work hours or retire early, resulting in significant productivity losses. The magnitude of these productivity losses is correlated with symptom severity and varies by country.

## Introduction

Myasthenia Gravis (MG) is a rare chronic autoimmune disorder associated with fatigue and weakness in the ocular, respiratory, bulbar and limb muscles (1). Patients not only incur healthcare costs, but also non-healthcare and informal care costs, related to the reduction in an individual’s capacity to carry out work and earn a living (2). The costs associated with reduced health-related productivity are commonly referred to as productivity losses. Productivity losses typically comprise of absenteeism (sick leave/being absent from work), presenteeism (individual presents to work, but their productivity is reduced while working) or stopping work altogether (early retirement or long-term disability leave) (3).

The symptoms of MG can severely affect an individual’s ability to perform daily activities, with multiple studies documenting the negative impact of MG on patients’ labor market experience, from changes to work and income to increased sick leave (2, 4–7). Patients with a disease or disability that affects their capability to carry out work or perform daily activities often rely on a close friend or family member to take on the role of caregiver to support them (8). This, in turn, can cause productivity losses in the caregiver, as they themselves reduce working hours or stop working altogether (9).

It has been estimated that approximately one third of MG patients require regular care from an informal caregiver, usually their spouse, family members or friends (2). However, there is limited research quantifying the productivity losses associated with caregiving in MG. A systematic literature review conducted by Landfeldt et al. found only one article that examined the informal costs of MG incurred by both patients and caregivers (10), indicating a clear need for additional research. Previous studies in other diseases have shown an association between disease severity and the caregiver’s ability to work in paid employment (11), hence, we hypothesize that a similar correlation might also exist between MG symptom severity and productivity losses for both caregivers and patients.

The objective of this study was threefold: to investigate potential reduced work productivity in patients and caregivers due to MG; to calculate the economic costs associated with this reduced productivity; and to illustrate how these costs can be used in economic evaluation.

## Materials and methods

To estimate reduced work productivity in patients and caregivers resulting from MG, we have considered data on sick leave, reduced working hours and stopping work reported in the MyRealWorld-MG study. In addition, we have evaluated the association between reduced work productivity and MG symptom severity, reported in the same time window. Per the human capital approach (12, 13), we calculated the costs resulting from this reduced work productivity by applying average wage costs on the findings for all respondents combined. For this, we took a UK perspective. In addition to the analyses performed on the whole sample, we conducted sensitivity analysis for countries with a sufficient sample size (> 100 people). In an applied example, to demonstrate how these productivity data can be used in economic evaluation, we explored the impact of improved symptom control on the productivity costs, by applying efficacy data from the ADAPT RCT study.

### Datasets

MyRealWorld-MG is a digital, observational study that began gathering real-world evidence on the burden of MG through patient-reported outcome data in December 2019. The study recruited 2,424 adult patients diagnosed with MG through clinical centers and/or patient advocacy groups across ten countries (US, UK, Canada, Italy, Germany, Spain, France, Belgium, Denmark, Japan). Using a smartphone application, patients provided data on demographics, disease duration, sick leave and caregiver help. They also completed the Myasthenia Gravis Activities of Daily Living (MG-ADL) and Myasthenia Gravis Foundation of America (MGFA) questionnaires (14). It should be noted that our analysis solely utilized data from MyRealWorld-MG entered at baseline. All patients were eligible for inclusion in the study. There were no exclusion criteria based on disease severity. The sole exception was that patients above the age of 65 were excluded from all analyses since the intention was to examine productivity losses related to paid work time, and it was assumed that patients above this age were more likely to be retired.

The MG-ADL consists of 8 items (talking, chewing, swallowing, breathing, brushing teeth and combing hair, rising from a chair, double vision and eyelid droop) across 4 domains (bulbar, respiratory, limb weakness and ocular). The 8 items are scored on a scale of 0 to 3 with the final cumulative score ranging between 0 and 24. Higher scores indicate the patient is suffering from more severe symptoms (15). We used the MG-ADL total score as a continuous variable in our analysis to estimate the association between symptom severity and reduced work productivity, and to categorize patients as having mild (0–4), moderate (5–9), or severe (> = 10) MG. This categorization matched previous MG-related investigations, followed the advice of neurologists, and mirrored the inclusion criteria from clinical trials, where a score of 5 or higher was used to classify patients as moderate to severe (14, 16, 17). The MGFA consists of 5 classes and several subdivisions that are used to group patients with similar clinical features or disease severity. The classes progress in severity, with Class I denoting exclusively ocular MG, while Class V refers to intubation with/without mechanical ventilation (18).

ADAPT (clinicaltrials.gov: NCT03669688) was a multi-center, double-blind, randomized controlled trial evaluating the efficacy of efgartigimod in adult patients with generalized MG (16). The primary outcome measure in this study was the MG-ADL score, which was measured at baseline and after 4 weeks of treatment. Unlike the MyRealWorld-MG study, which recruited patients from all severity levels, ADAPT only enrolled patients with moderate to severe generalized MG. To apply the relationship between MG-ADL and productivity losses observed in the MyRealWorld-MG dataset in a cost analysis, we have utilized the change in MG-ADL between baseline and 4 weeks of treatment with efgartigimod or standard of care (SoC).

### Statistical analysis

We evaluated productivity losses based on the latest available data cut from MyRealWorld-MG study at the time of analysis (July 2023). In the base case analysis, we included data from all countries. Productivity losses were firstly analyzed and reported by symptom severity, using the MG-ADL score. Next, reductions in productivity were examined by disease duration to understand changes in types of productivity losses over time. Thirdly, logistic regression models were developed to evaluate the impact of patient characteristics on work productivity for key outcomes of interest: whether sick leave was taken in the past month (yes/no), whether patients needed regular help from a caregiver (yes/no), and whether caregivers stopped paid work or reduced their working hours (yes/no). In all three equations, the explanatory variables considered were based on determinants found in published literature. These variables included MG type, patient age, sex, geographical region (Europe, Japan, US + Canada) and disease duration. *p*-values from a Wald Chi-Square test and Type-3 *p*-values are reported, with the former testing the significance of individual response categories against the reference category, and the latter if the inclusion of the predictor variable in the model significantly improves the fit compared to a model without that predictor. Data were analyzed using SAS version 9.4 (2020), proc genmod with a binary distribution and a logit link.

### Cost analysis

Our base case cost analysis was based on the whole MyRealWorld-MG dataset and took a UK perspective. We estimated productivity losses by multiplying the number of working hours lost per month (sick leave, reducing hours or stopping work) by the average wage per hour in the United Kingdom (UK) (19), calculated to be £20.44/h using 2023 data. The number of hours per lost workday were calculated to be 7.28. Mean monthly productivity losses were extrapolated over 1 year to estimate annual productivity losses, separately according to each MG-ADL score (0–24) and overall. In sensitivity analysis, productivity loss estimates for three countries were estimated in a comparable fashion using their country-specific MyRealWorld-MG data on wages, worktime and reductions in work productivity, combined with OECD average wage estimates for the country in question (20–22). The selected countries were Italy, the United States and Spain, as they had, in that order, the highest number of patients, jointly comprising 69.2% (726 patients) of the total sample.

As an example of how these productivity loss estimates can be applied, we combined these data with clinical efficacy data to estimate the change in productivity losses associated with treatment. In this study, we used the distribution of patients by MG-ADL at study baseline and week 4 from the ADAPT trial to explore the impact efgartigimod may have on work productivity losses (16).

## Results

### Patient characteristics

Of the 2,424 patients from the MyRealWorld-MG study, 1,049—those with sick leave and MG-ADL data and/or caregiver and MG-ADL data—were included for this analysis (Table 1). These patients were predominantly women (73.4%), with the majority (52.3%) aged between 35 and 54 years. They had been diagnosed with MG on average 8 (Standard Deviation (SD) 9.8) years previously. The sample included patients from 10 countries, with most being from Italy (32.8%), the United States (24.3%) and Spain (12.1%). The same trends were observed in the total MyRealWorld-MG population (Supplementary Table S1).

**Table 1:** Patient characteristics, with data on work productivity.

Patients		*N*
Sample sizes	All MyRealWorld-MG patients	2,424
	Of those, patients who are 18–65 years	2,074
	Of those, patients with data on sick leave & MG-ADL, and/or with data on the need of a caregiver & MG-ADL	1,049

Patients		Proportion of *N* = 1,049
MGFA	I: Ocular	13.4%
	II: Mild generalized	28.9%
	IIIa, IIIb: Moderate generalized	39.0%
	IV: Severe generalized	17.2%
	V: Intubation/Myasthenic crisis	1.5%
Time since diagnosis	Years (SD)	8 (9.8)
MG-ADL category	Mild: 0–4	42.3%
	Moderate: 5–9	39.9%
	Severe: 10–24	17.8%
Gender	Female	73.4%
Age	18–34	21.6%
	35–54	52.3%
	55–65	26.1%
	Above 65 (assumed to be retired)	0%
Country	Belgium	3.3%
	Canada	1.6%
	Denmark	2.4%
	France	4.7%
	Germany	8.2%
	Italy	32.8%
	Japan	7.8%
	Spain	12.1%
	UK	2.8%
	US	24.3%

MG, Myasthenia Gravis, MG-ADL, Myasthenia Gravis-Activities of Daily Living scale, MGFA, Myasthenia Gravis Foundation of America clinical classification, SD, standard deviation.

### Reduced work productivity due to sick leave and reduced working hours among MG patients

MyRealWorld-MG data indicated that patients’ capacity to work was severely affected by MG (Table 2). Absenteeism data indicated that 36.5% of patients had taken sick leave in the past month, with an average of 14.5 days lost per month. In addition, 11.4% of patients indicated they had not taken sick leave because the severity of their illness prevented them entirely from working, or they had retired early. The study did not include questions on presenteeism for MG patients and thus yielded no data on this front.

**Table 2:** Work productivity among MG patients.

Total sample	*N* = 1,049
MG patients with data on sick leave	*N* = 967
% patients did take time off work/studies in the past month	36.5%
Average number of days:	14.5
(SD, Q1–Q3)	(11.9, 3–30)
% patients who cannot work/study or retired early because of MG	11.4%
Average number of days:	22
% Did not take time off work/studies in the past month for the following reasons	52.1%
I have not taken sick leave	45.2%
I am retired (not because of MG)	3.1%
Other	2.4%
I choose not to work/study	0.9%
I am on leave	0.4%
I cannot work because of a disability other than MG	0.1%
I am unemployed at this time but not because of MG	0.0%

MG, Myasthenia Gravis, SD, standard deviation, Q1/Q3, first/third quartile.

### Reduced work productivity due to reduced working hours among caregivers

In MyRealWorld-MG, the percentage of patients requiring assistance from a caregiver when carrying out their regular activities was 36.0% (Table 3). In almost all cases, this caregiver was a family member or partner. On average, a caregiver dedicated 1.7 (SD 1) h a day to caregiving, which affected the amount of time they could dedicate to their work. In total, 14.6% of caregivers reported cutting working hours, with an average of 13 h cut per week, while 15.5% had to stop working altogether.

**Table 3:** Work productivity reductions among caregivers of MG patients.

Total sample	*N* = 1,049
MG patients with data on need for a caregiver	*N* = 999
Need help with regular activities from a caregiver	
Yes	36.0%
No	64.0%
Among those who have indicated they needed a caregiver:	*N* = 360
Different types of caregivers	
Family member/partner	96.1%
Friend	20.3%
Nurse or healthcare assistant	7.9%
Neighbour	10.2%
Community/Religious organization	2.0%
Domestic/Cleaning help	24.5%
Colleague	4.1%
Other	6.5%
Hours of help needed from all caregivers per day	*N* = 360
Mean (SD)	1.7 (1)
Hours of help needed from all caregivers per week	*N* = 360
0–7	49.3%
8–14	25.4%
15–49	18.6%
50 +	6.8%
Impact on the primary caregiver’s work:	*N* = 356
Had to cut down work	14.6%
Average hours cut down per week	13.0
Had to stop working	15.5%

MG, Myasthenia Gravis, SD, standard deviation.

### Reduced work productivity by MG symptom severity (MG-ADL score)

**Figure 1:**
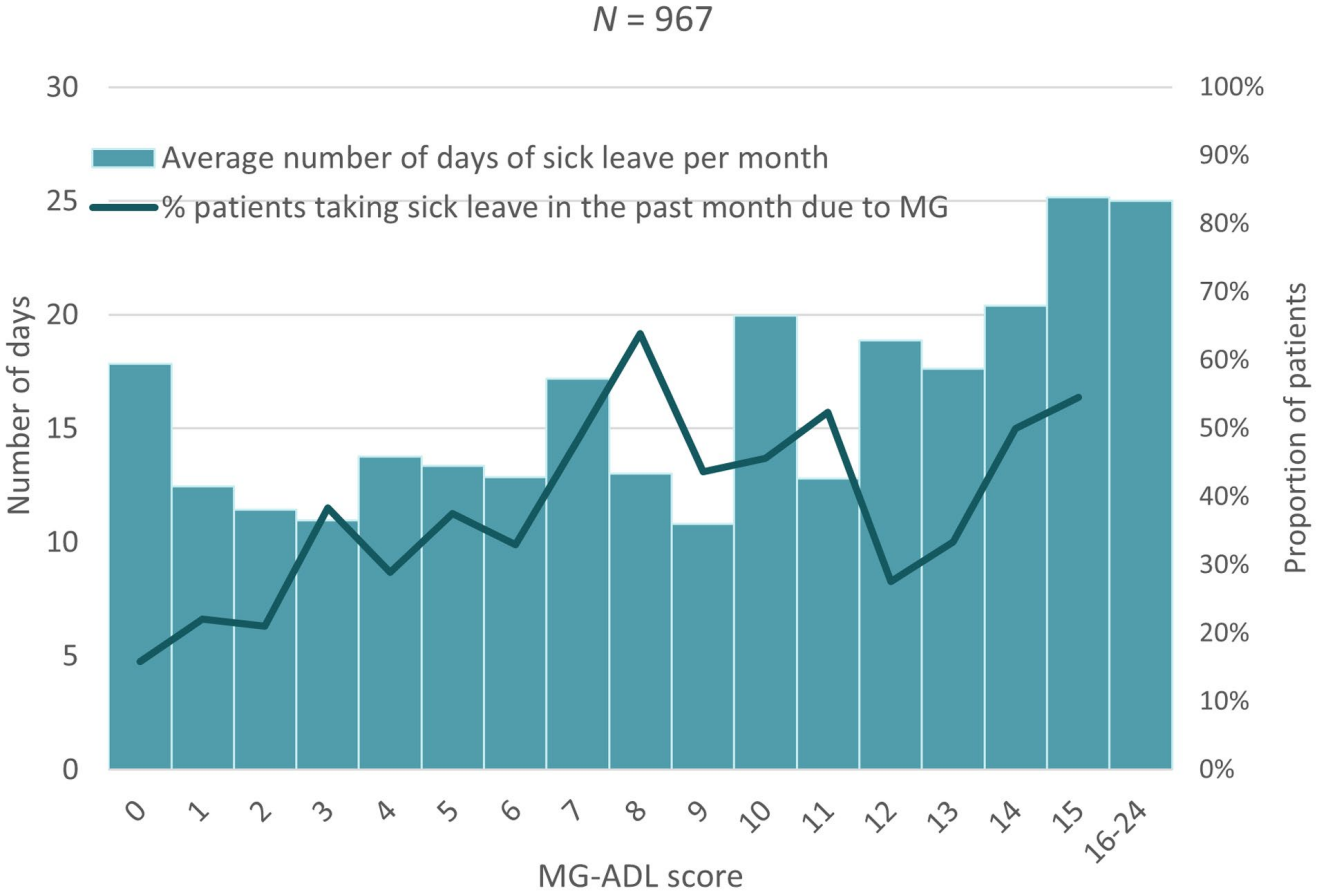
Time off work by MG-ADL score.

**Figure 2:**
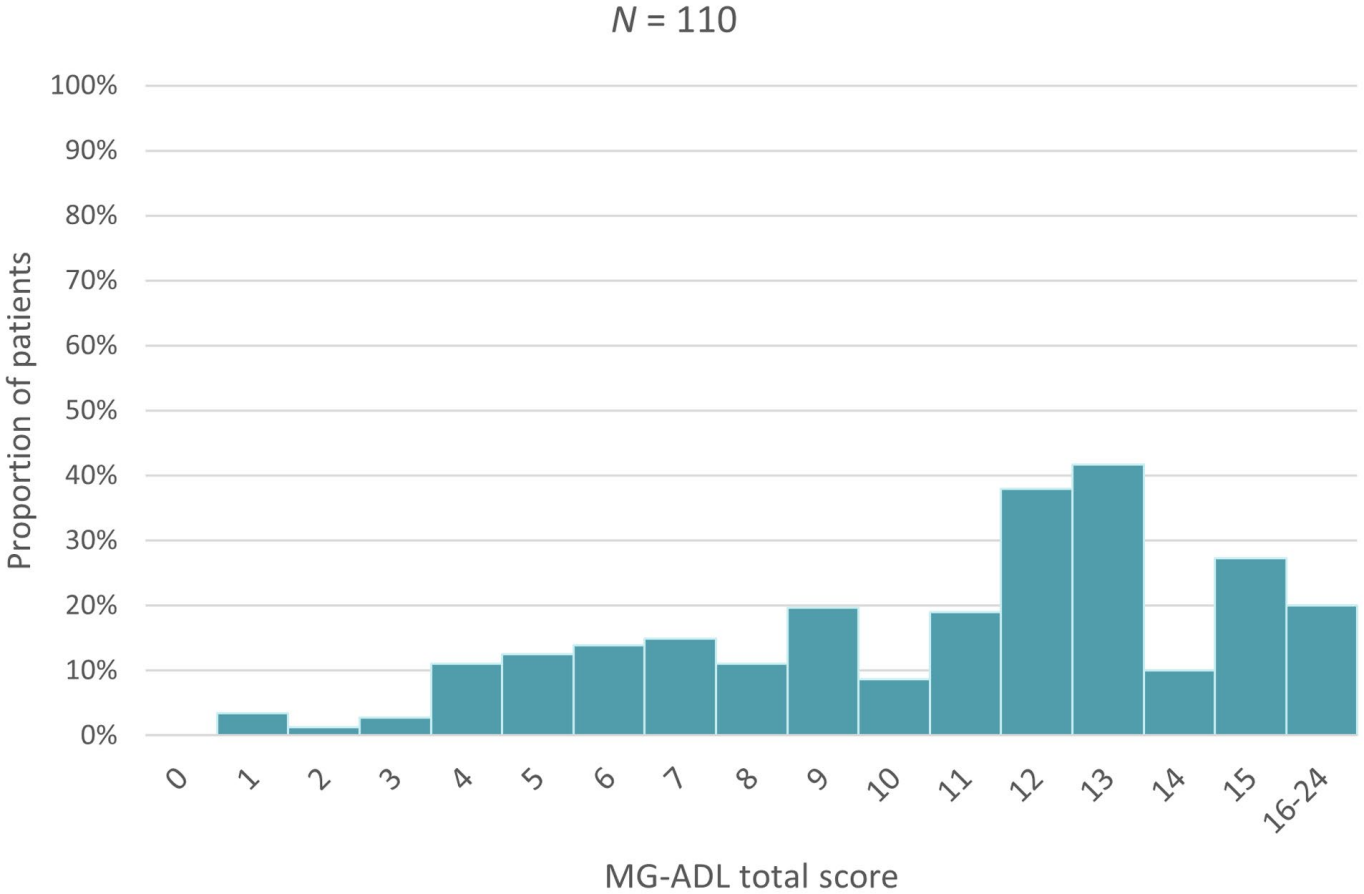
Proportion of patients retiring early or unable to work because of MG.

**Figure 3:**
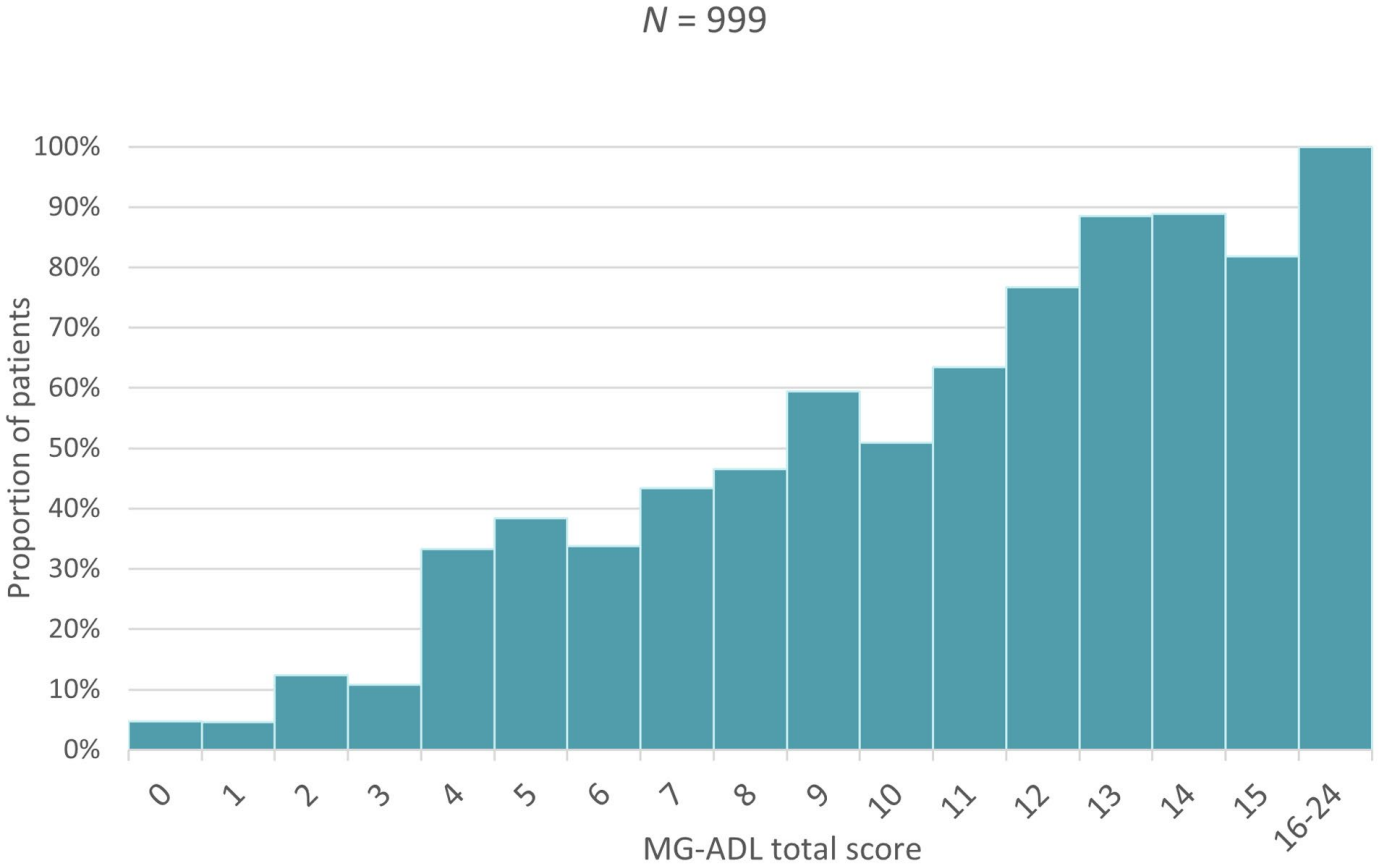
Proportion of patients requiring caregiver help.

**Figure 4:**
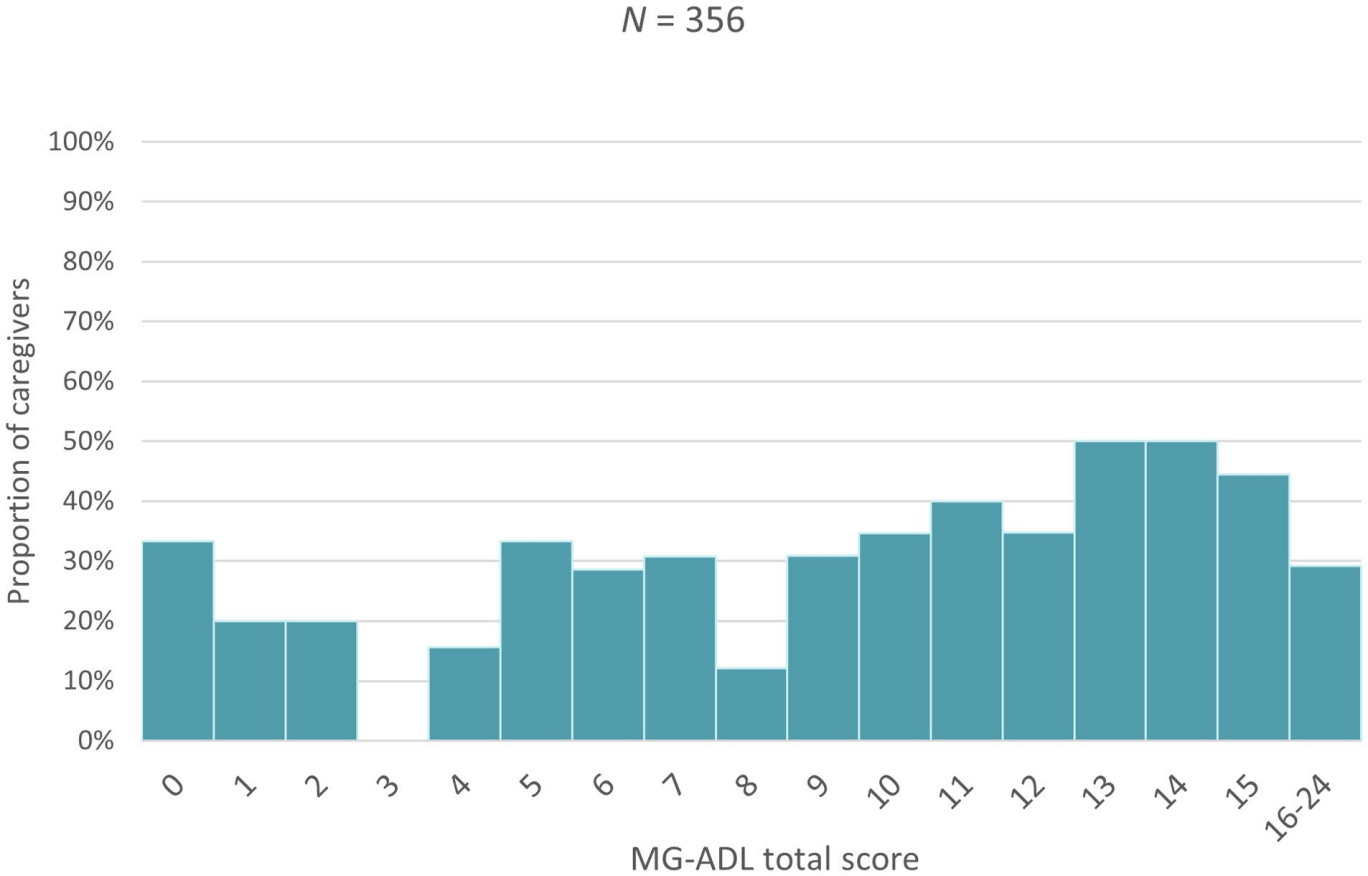
Proportion of caregivers reducing work hours or stopping paid work.

Higher MG-ADL scores (i.e., more severe disease) were associated with a higher proportion of patients taking sick leave, an increase in the number of sick days taken as well as a greater proportion of patients needing assistance in carrying out daily activities (Figures 1–3). Higher MG-ADL scores also were associated with the inability to work. The proportion of caregivers reducing working hours or stopping paid work increased slightly as a result of higher MG-ADL scores (Figure 4).

### Type of work productivity reductions by disease duration

Patients were most likely to take time off from work in the form of sick leave within the first 2 years after their diagnosis (Table 4). As disease duration increased, the proportion of patients taking sick leave decreased, while the proportion of patients going on disability leave or retiring early increased. The highest proportions of patients no longer being able to work due to their disease were observed in the groups that had lived 5–10 and 11–20 years with MG (16.8 and 15.3% respectively). Caregivers also increasingly stopped working or retired early as the duration of MG increased and they had to assist one third or more of patients throughout the follow-up.

**Table 4:** Observed differences in the type of work productivity reductions among non-retired people suffering from MG by disease duration.

MG patients with data on disease duration		Patients			Caregivers		
Disease duration (years)	*N* = 911, %	% Did take time off work/studies in the past month due to MG (*N* = 822)^*^	Average number of days of sick leave among patients with sick leave (*N* = 302)^*^	I cannot work/study or I retired early because of MG (*N* = 822)^*^	% Patients needing caregiver help (*N* = 871)^*^	Reduced working hours due to caregiving (*N* = 306)^*^	Stopped working or retired early because of caregiving (*N* = 306)^*^
<1 year	9.99%	59.8%	18.71	6.1%	35.6%	29.0%	9.7%
1 year	18.88%	52.0%	17.19	3.9%	37.5%	12.9%	11.3%
2–4 years	24.81%	39.5%	12.44	11.7%	35.3%	14.3%	13.0%
5–10 years	19.21%	32.3%	11.49	16.8%	35.5%	13.6%	15.3%
11–20 years	15.92%	24.4%	10.73	15.3%	39.1%	22.2%	24.1%
>20 years	11.20%	16.9%	9.00	11.2%	24.5%	8.7%	30.4%

* Decreased sample size due to missing responses on questions regarding work time lost/sick leave and disease duration. MG, Myasthenia Gravis.

### Patient and disease characteristics associated with productivity reductions

All three multivariable regression equations indicated that increases in the MG-ADL score significantly raised the odds that patients would take sick leave, that they would need help from a caregiver, and that the caregiver would need to reduce working hours (Supplementary Tables S2–S4). Multivariable regression analyses also revealed that time since diagnosis, patient age and region were associated with taking sick leave: older patients were less likely to take sick leave, while recently diagnosed patients and patients from Japan were more likely to take sick leave. Similarly, patient age and region were associated with needing a caregiver: patients between 30–39 years-old were more likely to require caregiving, while patients from Japan were less likely to seek the assistance of a caregiver (Supplementary Tables S2, S3). It is noted that these analyses utilize pooled data from different countries/regions, which could have affected the resulting *p*-values. Sensitivity analyses regarding MG type (ocular vs. generalized) were also conducted, but no statistically significant associations with taking sick leave, needing a caregiver or caregivers reducing working hours were found.

### Calculation of productivity losses by MG-ADL category

Table 5 shows that total productivity losses based on all patients in the MyRealWorld-MG cohort and on UK wage estimates amounted to £16,630, of which £13,891 (84%) was incurred by patients and £2,739 (16%) was incurred by caregivers. The majority of productivity losses were the result of taking sick leave, while caregivers primarily incurred productivity losses as a result of retiring early. A clear association between MG symptom severity and productivity losses emerged from our analysis of this dataset: the total productivity losses per year on average for patients with mild MG was £7,884 compared to £19,897 for patients with moderate MG and £30,016 for patients with severe MG. Patients with severe MG incurred 3.2 times more productivity losses compared to patients with mild disease, while the caregivers of patients with severe MG incurred 11.9 times more productivity losses than the caregivers of patients with mild MG.

**Table 5:** Average productivity losses by MG-ADL score.

	Patients					Caregivers					
MG-ADL	Sick days due to MG per year (mean)	Stop working/early retirement due to MG: days lost per year (mean)	Productivity losses per year for sick leave due to MG per patient^*^	Productivity losses due to stopping work/early retirement due to MG^*^	TOTAL productivity losses associated with reduced working by patients	Lost working hours per year by caregivers due to working fewer hours	Stop work/early retirement days lost per year (mean)	Productivity losses for caregivers due to working fewer hours^*^	Productivity losses due to stop working/early retirement^*^	TOTAL productivity losses associated with reduced working for caregivers	Grand total
All Patients (weighted average by individual MG-ADL score)	63.34	30.03	£9,423	£4,468	£13,891	32.47	13.95	£664	£2,076	£2,739	£16,630
By symptom severity											
Mild	38.8	10.4	£5,774	£1,544	£7,319	6.2	3.0	£126	£439	£566	£7,884
Moderate	73.8	38.0	£10,977	£5,654	£16,631	32.8	17.4	£670	£2,596	£3,267	£19,897
Severe	98.0	58.7	£14,574	£8,729	£23,303	94.3	32.2	£1,927	£4,787	£6,714	£30,016

* Calculated wage per hour (£20.44) multiplied by total number of work hours lost (average per workday: 7.28 h). MG, Myasthenia Gravis, MG-ADL, Myasthenia Gravis-Activities of Daily Living scale.

Furthermore, the proportion of caregiver productivity losses (as part of the total losses) increased with more severe disease: it was around 7.2% for patients with mild disease compared to 22.4% of total productivity losses for caregivers of severe MG patients.

### Sensitivity analysis by country

Whilst the base case analysis included data from all 10 countries in MyRealWorld-MG, this sensitivity analysis reports country-specific data from the countries with sufficient data to analyze results by MG-ADL, namely Italy, the US and Spain (Supplementary Table S5). Reductions in patient and caregiver productivity in Italy and Spain followed a similar trend as the overall data from MyRealWorld-MG: approximately a third of patients reported taking sick leave in the last month and approximately a third reported requiring caregiver support. In contrast, productivity reductions in the US were reported to be higher than other countries with approximately 45% of patients reporting taking sick leave in the last month and 49% requiring caregiver support. In all three countries, a large proportion of caregivers had to reduce working hours or stop working altogether, although very few patients were found to retire early. It is noted that in absolute value the productivity losses will be quite different between these countries, due to a different structure of productivity losses, determined by the country’s labor laws, social security and access to healthcare, and a different absolute prevalence of patients.

Our analyses also showed a clear association between MG symptom severity and productivity costs in Italy, Spain and the US (Table 6), although estimates showed considerable between-country variability, attributable to wage differences on the one hand, and variability in the proportions of sick leave and early retirement on the other hand. Notably, productivity losses for caregivers of severe MG patients in Italy were markedly high compared to the other countries, comprising up to 42% of the total productivity losses experienced by severe MG patients and their caregivers. Spain showed the highest total productivity losses for MG patients per year out of the three countries examined. In the United States, total productivity losses per year correlated the least with MG symptom severity.

**Table 6:** Comparison of productivity losses across countries.

Country	MG-ADL category	Patient Productivity Losses	Proportion	Caregiver Productivity Losses	Proportion	Grand total in local currency	Currency Adjusted to €
UK	Mild	£7,319	93%	£566	7%	£7,884	9,201 €
	Moderate	£16,631	84%	£3,267	16%	£19,897	23,222 €
	Severe	£23,303	78%	£6,714	22%	£30,016	35,030 €
	All patients	£13,891	84%	£2,739	16%	£16,630	19,408 €
Italy	Mild	4,297 €	95%	213 €	5%	4,510 €	4,510 €
	Moderate	10,425 €	72%	4,082 €	28%	14,507 €	14,507 €
	Severe	16,622 €	58%	11,922 €	42%	28,544 €	28,544 €
	All patients	8,021 €	73%	3,033 €	27%	11,055 €	11,055 €
Spain	Mild	10,888 €	94%	674 €	6%	11,562 €	11,562 €
	Moderate	13,860 €	83%	2,749 €	17%	16,608 €	16,608 €
	Severe	18,958 €	76%	5,934 €	24%	24,892 €	24,892 €
	All patients	13,167 €	86%	2,221 €	14%	15,388 €	15,388 €
US	Mild	$8,160	86%	$1,302	14%	$9,462	8,704 €
	Moderate	$9,063	72%	$3,541	28%	$12,604	11,594 €
	Severe	$10,997	74%	$3,791	26%	$14,788	13,604 €
	All patients	$9,468	75%	$3,141	25%	$12,609	11,600 €

MG-ADL, Myasthenia Gravis-Activities of Daily Living scale.

### Exploration of reductions in productivity losses due to improvements in MG-ADL: efgartigimod versus SoC

**Figure 5:**
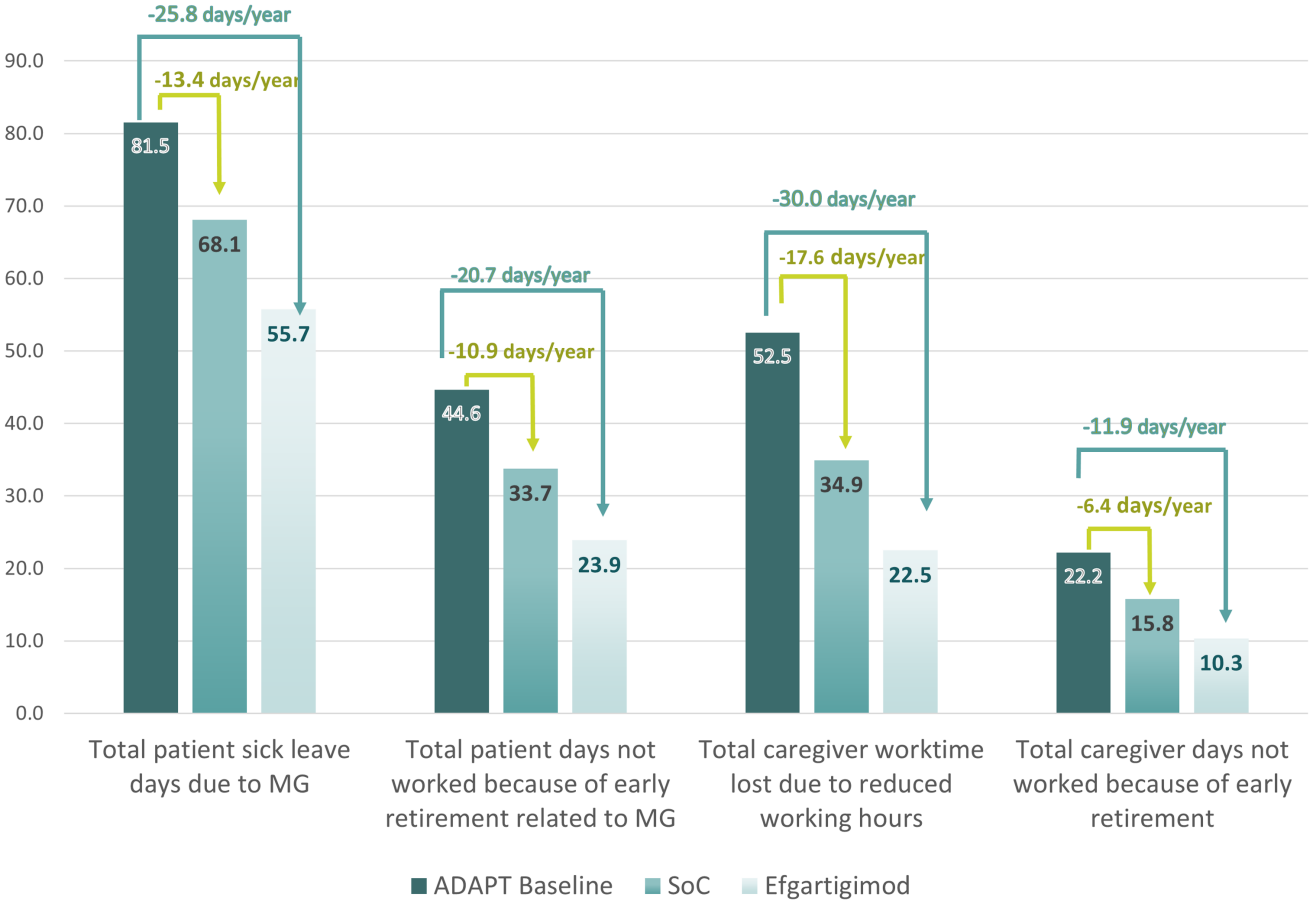
Estimated reduction in days per year not worked by patients and their caregivers due to MG.

**Figure 6:**
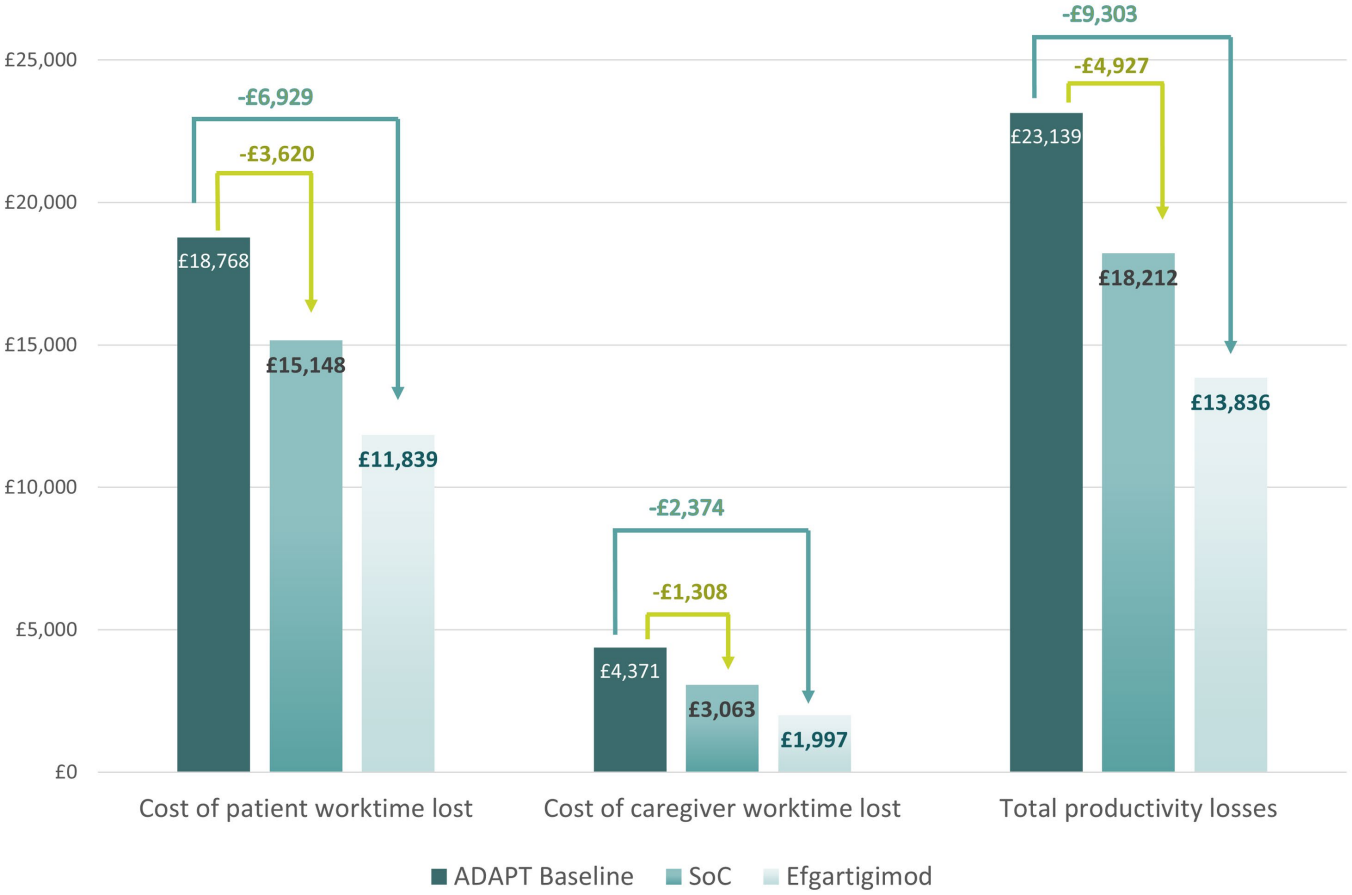
Projected yearly reductions in productivity losses associated with the use of efgartigimod.

Table 7 shows an example cost analysis that utilizes the MG-ADL distribution reported in the ADAPT study at baseline. As the goal of the study was to evaluate the efficacy of efgartigimod, only patients with moderate-to-severe MG were enrolled, hence the lack of patients with mild disease severity at baseline. When the disease severity distribution after 4 weeks in the SoC arm is compared with that of the treatment arm, a clear treatment effect can be observed: a little over a quarter of patients in the SoC arm reported mild disease severity (27%), compared to over half of patients in the treatment arm (57%). Using data on productivity losses (previously reported in Table 5) and the severity distributions from ADAPT, we calculated the impact of efgartigimod on productivity losses with a weighted average. Productivity losses were estimated to fall from £23,139 to £13,836 for efgartigimod treated patients (a 40.2% reduction in costs), compared to £18,212 for SoC patients (a 21.3% reduction in costs). Our analysis suggests that efgartigimod treatment could reduce the number of work days lost by patients and caregivers, mitigating productivity losses by £4,376 compared to SoC and £9,303 versus baseline (Figures 5, 6). This difference in productivity losses of £4,376 could be considered a gain in productivity.

**Table 7:** Example cost analysis: reduced productivity losses due to improvements in MG-ADL after 4 weeks of treatment with efgartigimod.

Results			
	Mild	Moderate	Severe
MG-ADL Category			
MG-ADL score distribution	0–4	5–9	10–24
At ADAPT baseline	0%	68%	32%
Standard of Care, after 4 weeks	27%	58%	15%
Efgartigimod, after 4 weeks	57%	35%	8%
Patients			
Days lost per annum for sick leave due to MG	38.8	73.8	98.0
Days lost per annum due to stopping work/early retirement due to MG	10.4	38.0	58.7
Productivity losses per year for sick leave due to MG per patient	£5,774	£10,977	£14,574
Productivity losses per year due to stopping work/early retirement due to MG per patient	£1,544	£5,654	£8,729
Total productivity losses per year	£7,319	£16,631	£23,303
Caregivers			
Days lost per annum due to reduced working hours	6.2	32.8	94.3
Days lost per annum due to stopping work/early retirement	3.0	17.4	32.2
Productivity losses per year for caregivers due to working fewer hours	£126	£670	£1,927
Productivity losses per year due to stopping working/early retirement	£439	£2,596	£4,787
Total productivity losses per year	£566	£3,267	£6,714
Grand Total	£7,884	£19,897	£30,016

Impact of efgartigimod on productivity losses	At Baseline, both treatments	SoC @ 4 weeks	Efgartigimod @ 4 weeks
Cost of patient worktime lost	£18,768	£15,148	£11,839
Cost of caregiver worktime lost	£4,371	£3,063	£1,997
Total productivity losses	£23,139	£18,212	£13,836
Change from BL		−£4,927	−£9,303
Impact of efgartigimod		−£4,376	

MG-ADL, Myasthenia Gravis-Activities of Daily Living scale, MG, Myasthenia Gravis, SoC, Standard of Care, BL, Baseline.

## Discussion

This study aimed to shed light on the societal impact of MG and evaluate the reduction in work productivity incurred by patients and their caregivers. MyRealWorld-MG evidence suggests that MG has a significant impact on work productivity for patients and caregivers, with patients taking a substantial amount of sick leave or giving up work altogether. Caregivers were similarly impacted, reducing working hours or quitting work altogether for their caregiving duties.

MG symptom severity measured by the MG-ADL score, was positively associated with an increasing proportion of patients taking sick leave and needing help from a caregiver. MG severity was also strongly associated with caregiver burden. Productivity losses were on average £16,630 per year for patients and caregivers combined, with severe MG patients incurring 3.2 times the productivity losses incurred by mild MG patients. It should be noted, however, that these numbers only capture the easily quantifiable fraction of the overall economic impact of MG on patients and their caregivers. Other aspects such as healthcare costs not covered by insurance, nonmedical costs and paid caregiving were not included.

In this study, we observed that the pattern of patient productivity losses appeared to evolve from sick leave to disability leave and early retirement. Over time, disease symptoms may have left patients unable to carry out previous levels of activity and they might have taken administrative steps to make their inability to carry out paid work permanent by registering for long-term disability.

Overall, our findings are consistent with previously published studies on the socio-economic burden posed by MG. In a large Japanese cohort, approximately one third of MG patients experienced unemployment or a decrease in income (7). An Australian study by the Centre for International Economics reported 42% of patients taking sick leave due to their symptoms; it also found that 39.4% of patients had stopped work due to MG and 19.4% had changed occupations (2). Twork et al. (23) and Lehnerer et al. (24) both carried out studies in the German population, with the former finding that 28.3% of patients had been forced to retire early (25) while the latter reported that 72.6% of patients had experienced limitations with regard to employment; of those patients, 45.8% ultimately stopped working (23).

The reductions in work productivity reported in this study are also in line with results from the MG-Quality of Life questionnaire (MG-QoL-15r) reported in the MyRealWorld-MG study (24), in which almost 38.4% of MG patients indicated that carrying out work or housework was “very much” a problem for them.

An inherent limitation of this study’s design is that patient data was collected entirely through the smartphone application MyRealWorld-MG and as a result, patients needed to have a smartphone and access to a Wi-Fi connection in order to take part. Furthermore, while patients were recruited through clinicians and patient advocacy groups, their diagnosis was self-reported in the application and could not be verified by cross-referencing to medical records. The patients recruited in this study are not necessarily an accurate reflection of the whole MG disease spectrum, which might introduce selection bias. In another multinational real-world study, the proportion of patients reporting ocular MG was larger, and the proportion reporting moderate-to-severe generalized MG was smaller (26). The same trend was observed for several smaller single-country studies (27–30). It is therefore possible that the productivity losses calculated in our study are an overestimation. Furthermore, patients with ocular problems might have experienced more difficulties in completing the smartphone application, which could also have introduced selection bias. Another limitation is that we used baseline data of this study to calculate productivity losses whereas the MG-ADL score is variable over time and therefore the productivity analysis may not accurately reflect the changes in severity of disease and in productivity losses over time. In addition, we did not take lost salary due to suboptimal work arrangement and other potential components of productivity losses as a result of MG into consideration during our calculation, potentially leading to an underestimation of the costs. A study reported substantial nonmedical costs and out of pocket expenses (supportive equipment; house/vehicle modifications such as stair lifts and automatic door openers; care for household members in the form of homeschooling or hiring a nanny; healthcare transportation and other travel-related expenses such as hotel costs and meals; schooling accommodations; mental health treatment/counselling; insurance premiums and prescription medicine) for both MG patients and their caregivers, none of which were captured in our calculations. Beyond working fewer hours, the study also described other ways in which patients and caregivers were occupationally affected, including missing opportunities for a better job, promotion or benefits, feeling a negative impact on their career growth, and being forced to switch to remote work (31). Finally, our cost analysis evaluating efgartigimod’s potential impact on work productivity utilized estimates of productivity losses from MyRealWorld-MG, which contained a different patient population compared to the ADAPT study. Thus, further research is needed to confirm these results.

This study illustrates a substantial socio-economic burden of MG, which is associated with a significant amount of lost work time for both patients and caregivers. Real-world evidence combined with clinical trial data suggest that any treatment demonstrating reduced symptoms may offer the promise of reducing productivity losses for both patients and caregivers. Understanding the wider societal impact of MG is a crucial and often overlooked aspect of the disease and is critical in designing rational patient focused interventions.

## Data Availability

The data analyzed in this study is subject to the following licenses/restrictions: Anonymized data are available upon reasonable request, and can be provided after review and approval of a research proposal and statistical analysis plan and execution of a data sharing agreement. Requests to access these datasets should be directed to sd@she-consulting.be.
