# Supplementary material for: A cost analysis of reductions in work productivity for MG patients and their caregivers by symptom severity
Source: Front Public Health. 2025 Apr 25;13:1538789. doi: 10.3389/fpubh.2025.1538789 (PMC12062151; doi:10.3389/fpubh.2025.1538789)
Supplement: Supplementary file 1 [file Table_1.docx]

**Table S1. Comparison of patient characteristics of the total MyRealWorld-MG study population and MyRealWorld-MG patients with data on productivity losses**

| **Patients** | | **Proportion of N=2424** | **Proportion of N=1049** |
| --- | --- | --- | --- |
| **MGFA** | I: Ocular | 15.5% | 13.4% |
|  | II: Mild generalized | 27.3% | 28.9% |
|  | IIIa , IIIb: Moderate generalized | 35.7% | 39.0% |
|  | IV: Severe generalized | 17.0% | 17.2% |
|  | V: Intubation / Myasthenic crisis | 1.3% | 1.5% |
| **Time since diagnosis** | Years (SD) | 8 (9.9) | 8 (9.8) |
| **MG-ADL category** | Mild: 0-4 | 42.7% | 42.3% |
|  | Moderate: 5-9 | 39.1% | 39.9% |
|  | Severe: 10-24 | 18.2% | 17.8% |
| **Gender** | Female | 67.0% | 73.4% |
|  | Male | 32.3% | 26.6% |
| **Age** | 18-34 | 17.1% | 21.6% |
|  | 35-54 | 43.7% | 52.3% |
|  | 55 - 65 | 39.2% | 26.1% |
|  | Above 65 (assumed to be retired) |  | 0% |
| **Country** | Belgium | 2.0% | 3.3% |
|  | Canada | 1.4% | 1.6% |
|  | Denmark | 3.0% | 2.4% |
|  | Germany | 5.4% | 8.2% |
|  | France | 2.4% | 4.7% |
|  | Italy | 36.3% | 32.8% |
|  | Japan | 6.9% | 7.8% |
|  | Spain | 9.4% | 12.1% |
|  | UK | 3.3% | 2.8% |
|  | US | 29.8% | 24.3% |
