# Supplementary material for: A cost analysis of reductions in work productivity for MG patients and their caregivers by symptom severity
Source: Front Public Health. 2025 Apr 25;13:1538789. doi: 10.3389/fpubh.2025.1538789 (PMC12062151; doi:10.3389/fpubh.2025.1538789)
Supplement: Supplementary file 2 [file Table_2.docx]

**Table S2. Impact of patient characteristics on taking sick leave in the past month**

| **Multivariable regression (n=817)** | **Description** | **OR** | **Wald Lower CI** | **Wald upper CI** | **P-value vs. reference category** | **P-value from Type 3 analysis** |
| --- | --- | --- | --- | --- | --- | --- |
| **MG-ADL score** | continuous | 1.09 | 1.05 | 1.13 | <.0001 | <.0001 |
| **Age** | 18-29 | 0.83 | 0.49 | 1.40 | 0.480 | 0.0053 |
|  | 30-39 | 0.72 | 0.45 | 1.13 | 0.155 |  |
|  | 40-49 (ref) | **ref** | **ref** | **ref** | **ref** |  |
|  | 50-59 | 0.83 | 0.55 | 1.26 | 0.386 |  |
|  | 60-65 | 0.34 | 0.19 | 0.60 | 0.000 |  |
| **Gender** | Female | 0.73 | 0.50 | 1.06 | 0.094 | 0.0937 |
|  | Male (ref) | **ref** | **ref** | **ref** | **ref** |  |
| **Region** | Europe | **ref** | **ref** | **ref** | **ref** | 0.0483 |
|  | Japan | 1.81 | 1.07 | 3.07 | 0.027 |  |
|  | US & Canada | 1.33 | 0.92 | 1.90 | 0.128 |  |
| **Duration** | Diagnosis <1 year ago | 3.00 | 1.69 | 5.31 | 0.000 | <.0001 |
|  | Diagnosis 1 year ago | 2.30 | 1.43 | 3.71 | 0.001 |  |
|  | Diagnosis 2-4 years ago | 1.34 | 0.85 | 2.11 | 0.202 |  |
|  | Diagnosis 5-10 years ago (ref) | **ref** | **ref** | **ref** | **ref** |  |
|  | Diagnosis 11-20 years ago | 0.64 | 0.37 | 1.10 | 0.105 |  |
|  | Diagnosis >20 years ago | 0.87 | 0.43 | 1.75 | 0.018 |  |
