# Supplementary material for: A cost analysis of reductions in work productivity for MG patients and their caregivers by symptom severity
Source: Front Public Health. 2025 Apr 25;13:1538789. doi: 10.3389/fpubh.2025.1538789 (PMC12062151; doi:10.3389/fpubh.2025.1538789)
Supplement: Supplementary file 3 [file Table_3.docx]

**Table S3.** **Impact of patient characteristics on needing help from a caregiver**

| **Multivariable regression (n=866)** | **Description** | **OR** | **Wald Lower CI** | **Wald upper CI** | **P-value from reference category** | **P-value from Type 3 analysis** |
| --- | --- | --- | --- | --- | --- | --- |
| **MG-ADL score** | continuous | 1.39 | 1.32 | 1.46 | <.0001 | <.0001 |
| **Age** | 18-29 | 0.80 | 0.44 | 1.43 | 0.443 | 0.0272 |
|  | 30-39 | 1.79 | 1.10 | 2.91 | 0.020 |  |
|  | 40-49 (ref) | **ref** | **ref** | **ref** | **ref** |  |
|  | 50-59 | 0.88 | 0.55 | 1.41 | 0.597 |  |
|  | 60-65 | 0.90 | 0.50 | 1.63 | 0.738 |  |
| **Gender** | Female | 1.26 | 0.81 | 1.96 | 0.300 | 0.3001 |
|  | Male (ref) | **ref** | **ref** | **ref** | **ref** |  |
| **Region** | Europe | **ref** | **ref** | **ref** | **ref** | <.0001 |
|  | Japan | 0.17 | 0.08 | 0.38 | <.0001 |  |
|  | US & Canada | 1.19 | 0.81 | 1.73 | 0.381 |  |
| **Duration** | Diagnosis <1 year ago | 0.88 | 0.46 | 1.70 | 0.709 | 0.3324 |
|  | Diagnosis 1 year ago | 1.43 | 0.83 | 2.44 | 0.197 |  |
|  | Diagnosis 2-4 years ago | 1.05 | 0.63 | 1.74 | 0.866 |  |
|  | Diagnosis 5-10 years ago (ref) | **ref** | **ref** | **ref** | **ref** |  |
|  | Diagnosis 11-20 years ago | 1.53 | 0.87 | 2.70 | 0.143 |  |
|  | Diagnosis >20 years ago | 0.44 | 0.22 | 0.87 | 0.694 |  |
