# Supplementary material for: A cost analysis of reductions in work productivity for MG patients and their caregivers by symptom severity
Source: Front Public Health. 2025 Apr 25;13:1538789. doi: 10.3389/fpubh.2025.1538789 (PMC12062151; doi:10.3389/fpubh.2025.1538789)
Supplement: Supplementary file 4 [file Table_4.docx]

**Table S4. Impact of patient characteristics on caregivers stopping work or reducing working hours**

| **Multivariable regression (n=303)** | **Description** | **OR** | **Wald Lower CI** | **Wald upper CI** | **P-value from reference category** | **P-value from Type 3 analysis** |
| --- | --- | --- | --- | --- | --- | --- |
| **MG-ADL score** | continuous | 1.10 | 1.02 | 1.17 | 0.010 | 0.0095 |
| **Age** | 18-29 | 1.32 | 0.55 | 3.20 | 0.534 | 0.9689 |
|  | 30-39 | 1.15 | 0.56 | 2.36 | 0.706 |  |
|  | 40-49 (ref) | **ref** | **ref** | **ref** | **ref** |  |
|  | 50-59 | 0.99 | 0.48 | 2.03 | 0.977 |  |
|  | 60-65 | 1.06 | 0.39 | 2.86 | 0.907 |  |
| **Gender** | Female | 0.88 | 0.42 | 1.84 | 0.740 | 0.7396 |
|  | Male (ref) | ref | ref | ref | ref |  |
| **Region** | Europe | **ref** | **ref** | **ref** | **ref** | 0.2224 |
|  | Japan | 2.17 | 0.58 | 8.14 | 0.252 |  |
|  | US & Canada | 0.72 | 0.41 | 1.27 | 0.257 |  |
| **Duration** | Diagnosis <1 year ago | 1.38 | 0.54 | 3.54 | 0.506 | 0.2525 |
|  | Diagnosis 1 year ago | 0.72 | 0.30 | 1.69 | 0.446 |  |
|  | Diagnosis 2-4 years ago | 0.94 | 0.43 | 2.05 | 0.881 |  |
|  | Diagnosis 5-10 years ago (ref) | **ref** | **ref** | **ref** | **ref** |  |
|  | Diagnosis 11-20 years ago | 1.92 | 0.85 | 4.32 | 0.114 |  |
|  | Diagnosis >20 years ago | 1.63 | 0.55 | 4.84 | 0.375 |  |
