# Supplementary material for: A cost analysis of reductions in work productivity for MG patients and their caregivers by symptom severity
Source: Front Public Health. 2025 Apr 25;13:1538789. doi: 10.3389/fpubh.2025.1538789 (PMC12062151; doi:10.3389/fpubh.2025.1538789)
Supplement: Supplementary file 5 [file Table_5.docx]

**Table S5. Comparison of productivity reductions across countries**

|  | **Italy** | | | **Spain** | | | **United States** | | |
| --- | --- | --- | --- | --- | --- | --- | --- | --- | --- |
|  | **Patients** | **Caregivers** | | **Patients** | **Caregivers** | | **Patients** | **Caregivers** | |
| **MG-ADL** | **% taking sick leave in the past month due to MG** | **% requiring CG help** | **% CG stopping work altogether *** | **% taking sick leave in the past month due to MG** | **% requiring CG help** | **% CG stopping work altogether *** | **% taking sick leave in the past month due to MG** | **% requiring CG help** | **% CG stopping work altogether *** |
| 0 | 23.3% | 2.2% | 0.0% | 14.3% | 16.7% | 0.0% | 16.7% | 0.0% | 0.0% |
| 1 | 5.3% | 2.8% | 0.0% | 33.3% | 8.3% | 0.0% | 33.3% | 12.5% | 100.0% |
| 2 | 12.9% | 2.8% | 0.0% | 22.2% | 10.0% | 100.0% | 50.0% | 20.0% | 0.0% |
| 3 | 40.9% | 0.0% | 0.0% | 43.8% | 28.6% | 0.0% | 36.4% | 0.0% | 0.0% |
| 4 | 32.0% | 32.1% | 0.0% | 25.0% | 44.4% | 0.0% | 37.5% | 22.2% | 25.0% |
| 5 | 28.6% | 25.0% | 40.0% | 23.5% | 26.3% | 20.0% | 52.6% | 64.7% | 18.2% |
| 6 | 39.4% | 51.5% | 29.4% | 20.0% | 18.2% | 0.0% | 31.6% | 27.3% | 0.0% |
| 7 | 44.0% | 42.3% | 18.2% | 27.3% | 36.4% | 50.0% | 44.0% | 46.2% | 16.7% |
| 8 | 60.0% | 47.1% | 12.5% | 66.7% | 40.0% | 0.0% | 60.0% | 56.5% | 8.3% |
| 9 | 25.0% | 68.4% | 0.0% | 85.7% | 42.9% | 0.0% | 45.5% | 62.5% | 13.3% |
| 10 | 38.5% | 57.1% | 37.5% | 50.0% | 50.0% | 0.0% | 53.3% | 50.0% | 11.1% |
| 11 | 33.3% | 77.8% | 42.9% | 0.0% | 100.0% | 50.0% | 65.0% | 60.0% | 0.0% |
| 12 | 20.0% | 75.0% | 33.3% | 0.0% | 100.0% | 0.0% | 25.0% | 72.7% | 12.5% |
| 13 | 50.0% | 66.7% | 80.0% | 0.0% | 100.0% | 0.0% | 30.0% | 100.0% | 15.4% |
| 14 | 0.0% | 100.0% | 0.0% | 100.0% | 100.0% | 0.0% | 66.7% | 80.0% | 0.0% |
| **Mild** | 20.8% | 6.8% | 0.0% | 30.8% | 21.5% | 17.3% | 36.7% | 12.9% | 20.4% |
| **Moderate** | 38.6% | 47.0% | 21.5% | 38.9% | 31.3% | 16.5% | 47.3% | 51.7% | 11.5% |
| **Severe** | 37.8% | 68.1% | 41.1% | 35.7% | 78.6% | 10.7% | 49.3% | 69.1% | 6.6% |
| **All patients** | **29.4%** | **28.9%** | **12.8%** | **35.0%** | **32.6%** | **16.2%** | **45.7%** | **48.8%** | **11.9%** |

*Assumed 22 days of productivity lost per month
